# Comparative analysis of CAR T-cell therapy access for DLBCL patients: associated challenges and solutions in the four largest EU countries

**DOI:** 10.3389/fmed.2023.1128295

**Published:** 2023-05-30

**Authors:** Miguel Á. Canales Albendea, Pier Luigi Canonico, Guillaume Cartron, Barthold Deiters, Claudio Jommi, Reinhard Marks, Catherine Rioufol, Juan M. Sancho Cia, Armando Santoro, Eva M. Wagner-Drouet

**Affiliations:** ^1^Department of Hematology, Clínica Universidad de Navarra, Pamplona, Spain; ^2^Department of Pharmaceutical Sciences, Università del Piemonte Orientale, Novara, Italy; ^3^Centre Hospitalier Universitaire de Montpellier, UMR-CNRS 5535, Montpellier, France; ^4^GWQ ServicePlus AG, Düsseldorf, Germany; ^5^Department of Medicine I, Medical Center, University of Freiburg, Freiburg, Germany; ^6^Department of Pharmacy, Lyon Sud Hospital, Hospices Civils de Lyon, Lyon, France; ^7^EA 3738 Center for Innovation in Cancerology of Lyon (CICLY)-Claude Bernard University Lyon I, Lyon, France; ^8^ICO-IJC-Hospital Germans Trias i Pujol, Badalona, Spain; ^9^Department of Biomedical Sciences, Humanitas University, Pieve Emanuele, Milan, Italy; ^10^IRCCS Humanitas Research Hospital, Humanitas Cancer Center, Milan, Italy; ^11^Department of Hematology and Oncology, University Medical Center, Mainz, Germany

**Keywords:** CAR T-cell therapy, diffuse large B-cell lymphoma, patient access, health system, Germany, France, Italy, Spain

## Abstract

**Introduction:**

CAR T-cell therapy has emerged as a promising new immuno-oncology treatment that engages the patient’s immune system to fight certain hematological malignancies, including diffuse large B-cell lymphoma (DLBCL). In the European Union (EU), CAR T-cell therapies have been approved for relapsed/refractory (R/R) DLBCL patients since 2018, but patient access is often still limited or delayed. This paper is aimed at discussing challenges to access and possible solutions in the largest four EU countries.

**Methods:**

The analysis relied on literature review, market data collection, since homogeneous data coming from registries were not available, and discussion with experts coming from all four countries.

**Results:**

We calculated that in 2020, between 58% and 83% of R/R DLBCL patients (EMA approved label population) or between 29% and 71% of the estimated medically eligible R/R DLBCL patients, were not treated with a licensed CAR T-cell therapy. Common challenges along the patient journey that may result in limited access or delays to CAR T-cell therapy were identified. These include timely identification and referral of eligible patients, pre-treatment funding approval by authorities and payers, and resource needs at CAR T-cell centers.

**Discussion:**

These challenges, existing best practices and recommended focus areas for health systems are discussed here, with the aim to inform necessary actions for overcoming patient access challenges for current CAR T-cell therapies as well as for future cell and gene therapies.

## Highlights

- Despite approval of CAR T-cell therapies in EU-4 countries, their use in relapsed/refractory DLBCL patients remains limited, with between 29 and 71% of estimated eligible patients not receiving treatment in 2020.
- Systematic challenges along the patient journey can limit or delay CAR T-cell therapy access for eligible patients, including identification, referral, pre-treatment funding approval and center resource needs.
- Local best practices and actionable recommendations presented in this study can guide health system efforts to improve patient access for current and future cell and gene therapies.

## 1. Introduction

### 1.1. CAR T-cell cancer immunotherapy developments

CAR T-cell therapies are novel anti-cancer treatments that utilize the immune system, specifically immune T-lymphocytes, or T-cells, to fight tumor cells. Patient T-cells are genetically modified to express chimeric antigen receptors (CARs), which target specific cancer cell-associated surface proteins. When infused back into the patient’s blood, CAR T-cells bind to cancer cells expressing these antigens and trigger a T-cell initiated cell destruction (1).

Six commercial CAR T-cell therapies have been approved by the U.S. Food and Drug Administration (FDA) and the European Medicines Agency (EMA) since 2017; four of them are designed to bind the B-lymphocyte antigen CD19 (Cluster of Differentiation 19) expressed on the cell surface of different types of lymphoma and leukemia, whereas the other two approved CAR T-cell therapies target the multiple myeloma-expressed B-cell maturation antigen BCMA (Supplementary Table 1). In 2020, EMA-approved CD19 CAR T-cell therapies covered the following indications: R/R diffuse large B-cell lymphoma (DLBCL) after at least two lines of therapy (2–4), with CAR T-cell therapy after only one line of chemoimmunotherapy approved by the EMA in October 2022 (5); R/R primary mediastinal large B-cell lymphoma after at least two lines of therapy (2, 4); R/R follicular lymphoma after at least two lines of therapy (2, 6); R/R mantle cell lymphoma after at least two lines of therapy including a Bruton’s tyrosine kinase inhibitor (7); and R/R B-cell acute lymphoblastic leukemia in pediatric patients up to 25 years after at least two lines of therapy or after relapse post-transplant (2). Indications approved by EMA for BCMA CAR T-cell therapies include R/R multiple myeloma after at least three lines of systemic therapy, including an immunomodulatory agent, a proteasome inhibitor and an anti-CD38 antibody and with demonstrated disease progression on the last therapy line (8, 9).

The growing number of approved CAR T-cell therapies is fueled by a rapidly expanding pipeline with more than 500 “active” CAR T-cell trials listed by the beginning of 2022 [“active” status corresponding to CAR T-cell studies listed as “*recruiting*,” “*enrolling by invitation*” or “*active, not recruiting*” on www.clinicaltrials.gov; (10)]. Ongoing research will likely increase the breadth of CAR immunotherapy applications and includes among others CAR T-cells targeting solid tumors, bispecific CAR T-cells targeting two different antigens, allogeneic “off-the-shelf” CAR T-cells and CAR NK (natural killer)-cells (1). Particularly for solid tumors, which constitute most of malignant neoplasms, CAR immunotherapy development may one day bring a much-needed novel treatment approach, akin to the successful application in different hematological cancers to date.

### 1.2. Access environment for DLBCL CAR T-cell therapies in EU-4 countries

In 2018/19, Germany, Italy and Spain granted reimbursement and commercial patient access for the CD19 CAR T-cell therapies *tisagenlecleucel* and *axicabtagene ciloleucel* in R/R DLBCL patients after at least two lines of therapy (11–15). France had already allowed patient access to *tisagenlecleucel* and *axicabtagene ciloleucel* before EMA market authorization, through its early access program (ATU), intended for therapies addressing a high unmet need without available alternatives [since 2021, the ATU/“*Temporary Authorization for Use*” program has been replaced by the APP/*“Early Access Authorization”* system (16)]. In 2019, both CAR T-cell therapies were approved by French authorities for statutory reimbursement listing and transitioned from the ATU program (17, 18). In the four European countries, integration of *tisagenlecleucel* and *axicabtagene ciloleucel* into care for R/R DLBCL patients was tightly controlled by the health authorities, including regulations for CAR T-cell center authorization, patient eligibility approval, data collection through registries and funding mechanisms (Table 1).

**Table 1:** DLBCL CAR T-cell therapy implementation mechanisms across EU-4 countries – CAR T-cell center authorization, patient eligibility criteria, treatment approval processes, data collection and funding mechanisms in France, Germany, Italy, and Spain.

Country	France	Germany	Italy	Spain
CAR T-cell center authorization *(in addition to center qualification by manufacturer)*	CAR T-cell center criteria defined at national level (19). Center authorization by regional health authorities (19).	CAR T-cell center criteria defined at national level (20). Center authorization by sick funds (and their medical review boards) (20).	CAR T-cell center criteria defined at national level (13, 14). Center designation, authorization by regional health authorities (13, 14).	CAR-T center criteria, designation, and authorization at national level (21, 22).
Patient eligibility criteria	Patient eligibility criteria defined by CAR T-cell center.	Patient eligibility criteria defined by CAR T-cell center. Restrictions beyond EMA label may be applied by sick funds (and their medical review boards) for CAR-T cell product funding, that are based on clinical trial criteria and are evolving with new clinical evidence. The eligibility criteria applied by the sick funds’ medical review boards can differ across German regions.	Patient eligibility criteria defined at national level. Restrictions beyond EMA label apply for CAR T-cell product funding, which are based on clinical trial criteria and additional 70 years age limit (increased to 75 years in May 2022 for *tisagenlecleucel*) (23–25).	Patient eligibility criteria defined at national level. Restrictions beyond EMA label apply for CAR T-cell product funding, which are based on clinical trial criteria (22).
Treatment approval	Center-level approval.	Pre-treatment approval by sick funds may be required/requested to minimize financial risks (26).	Center-level approval through completion of AIFA registry form with eligibility checklist (corresponding to an implicit authority approval) (23, 24). Additional pre-treatment approval may be required by the authorities of the region of patient origin.	Pre-treatment approval required by authorities of the region of patient origin and the national expert group (22, 27).
Registry for data collection *(in addition to EBMT registry requirement)*	National registry (DESCAR-T) (28). The registry is supporting yearly reassessment of approval based on real world effectiveness (17, 18, 29).	National registry (DRST) (26). In addition, data collection by sick funds is supporting outcome-based rebate agreements (29).	National AIFA registry (13, 14). The registry is supporting implementation of outcome-based staged payments (29).	National registry (VALTERMED) (27, 30). The registry is supporting implementation of outcome-based staged payments (29).
CAR T-cell product funding mechanisms	National funding for innovative, high-cost products through the “*Liste en Sus*” as well as the early access programs ATU/AAP (16, 29).	Sick fund-level funding, e.g., through application by the center for a NUB innovation funding (29).	National funding for innovative oncology products allocated to and managed by regional authorities (“*Fondo Farmaci Oncologici Innovativi*”) (13, 14, 29).	Regional-level funding.

#### 1.2.1. Center authorization and qualification

CAR T-cell therapies can be only provided by authorized CAR T-cell centers, that fulfil specific structural and organizational quality requirements defined by national authorities (13, 14, 19–21). In Spain, CAR T-cell centers were specifically designated by the Ministry of Health (22), and in Italy, due to the decentralized architecture of the health care system, by the respective regional authorities (13, 14), effectively authorizing only a sub-set of the centers that fulfill the quality criteria for active CAR T-cell therapy use. In France and Germany, centers fulfilling national criteria were authorized by regional health authorities or sick funds, respectively (20). In addition to authorization by authorities, pharmaceutical manufacturers have defined specific qualification procedures that centers need to complete before providing commercial CAR T-cell therapies.

#### 1.2.2. Patient eligibility criteria

Beyond the EMA-approved R/R DLBCL indication for *tisagenlecleucel* and *axicabtagene ciloleucel*, patient eligibility criteria were further restricted at national level in Germany, Italy and Spain based on the selection criteria applied in the registrational trials (22–24, 26). In Germany, although not officially published, criteria for CAR T-cell therapy reimbursement have been developed by the sick funds’ medical review boards under the guidance of the national Competence Center Oncology [KCO/*“Kompetenz-Centrum Onkologie”* (31)]. These patient eligibility criteria continue to evolve based on new clinical evidence, and their implementation by the sick funds’ medical review boards differs across German regions. In Italy, in addition to the registrational trial criteria, a maximum patient age of 70 years was defined for CAR T-cell therapy by authorities (increased to 75 years in May 2022 for *tisagenlecleucel*) (23–25). In France, no eligibility criteria for DLBCL CAR T-cell therapies beyond EMA regulatory label were defined at the national level to our knowledge.

#### 1.2.3. Pre-treatment approval

In France, patient eligibility for CAR T-cell therapy is assessed and confirmed at the discretion of the CAR T-cell center. In Germany, as long as no automatic funding process is implemented, sick funds supported by their medical review board decide on funding approval for each CAR T-cell therapy patient. Once automatic funding is established [via a NUB/“Neue Untersuchungs-und Behandlungsmethoden - New diagnostic and treatment method” funding for innovative medical procedures agreed at center level (29)], authorized centers can in theory provide CAR T-cell therapies within the label without pre-treatment approval by sick funds. However, due to the financial risks involved, centers may choose or be required by sick funds to collect this approval before use of the CAR T-cell therapy, despite existing NUB agreement (26). In Italy and Spain, authority approval is required for each patient before use of CAR T-cell therapy. In Italy, funding approval is sought by CAR T-cell centers through registration with the online AIFA registry platform (23, 24), but in addition, approval can also be required from the region of patient origin. In Spain, treatment approval must be given by the authorities of the region of patient origin as well as by a national expert group that confirms final eligibility for CAR T-cell therapy (27).

#### 1.2.4. Registries

In France, Italy and Spain, CAR T-cell centers are required to collect patient outcome data in national CAR T-cell registries, which in France are also used to reassess CAR T-cell therapy approval based on demonstrated real-world effectiveness (17, 18, 28, 29), and in Italy and Spain, support outcome-based staged payments with pharmaceutical manufacturers (13–15, 29, 30). In Germany, CAR T-cell centers report data to the German Registry for Stem Cell Transplantation (DRST) (26). In addition, data collection for outcome-based rebate agreements with pharmaceutical manufacturers is managed by sick funds directly (29). In addition to national registries, European centers are requested to participate in data collection for the European Society for Blood and Marrow Transplantation (EBMT) CAR T-cell registry, also supporting post-authorization safety studies mandated by the EMA (32).

#### 1.2.5. Funding mechanisms

Provided the above conditions are fulfilled, French and Italian authorized centers receive CAR T-cell product funding through a national financing mechanism for innovative, high-cost products (“*Liste en Sus*”/“*Supplementary List*” in France, “*Fondo Farmaci Oncologici Innovativi*”/“*Funds for Innovative Oncological Medicines*” in Italy) (13, 14, 29). Both in France and Italy, CAR T-cell product funding is conditioned by the centers providing information through the national registry to health authorities. In Italy, CAR-T cell product funding is allocated to the regions by the central government and managed by the regional authorities. In Spain, CAR T-cell product funding is managed at a regional level, and in Germany, CAR T-cell therapy funding *via* center-level NUB agreements is managed by the patient’s sick fund individually (29).

### 1.3. DLBCL patients journey to CAR T-cell therapy

Before CAR T-cell therapy is indicated, DLBCL patients must undergo a multi-step diagnostic and therapeutic journey. After initial DLBCL diagnosis, patients start with a first line chemoimmunotherapy, generally using the combination regimen R-CHOP (33). Recently, the POLARIX trial has demonstrated significantly better PFS (Progression Free Survival) for the combination Polatuzumab vedotin + R-CHP compared with standard R-CHOP (34). However, it remains to be defined whether it will be considered as a new standard, bearing in mind the slight difference in PFS, no clear benefit in certain subgroups, and mainly, the lack of difference in OS (Overall Survival) (35). Otherwise, a consequence drawn from this first analysis is the reduction of patients receiving subsequent CAR T-cell therapy (34). Use of other R-CHOP-like regimens is generally not supported as first-line treatment due to lack of evidence for better outcomes and/or their higher toxicity vs. standard R-CHOP.

Up to 50% of DLBCL patients will be refractory to first line therapy or will experience relapse after initial response, thereby requiring second line therapy, which generally consists of salvage chemotherapy, followed by consolidation with autologous stem cell transplantation for those eligible (33). Around 80% of DLBCL patients undergoing second line therapy will be refractory or eventually relapse (33), becoming eligible for CAR T-cell therapy per the EMA regulatory label (2, 3). Based on our analysis, this corresponds to 14–21% of DLBCL patients in the EU-4 countries assessed (Supplementary Table 2). It must be noted that the above treatment pathway reflects patient reality in 2020, before EMA label for *axicabtagene ciloleucel* has been extended in October 2022 for use in DLBCL patient that relapses within 12 months from completion of, or is refractory to, first-line chemoimmunotherapy (3), which is likely to change the patient treatment pathway from second line on.

Once a patient is identified as a potential candidate for CAR T-cell therapy by their treating hematologist, support for confirmation of treatment choice and eligibility ideally is sought directly from a CAR T-cell center or from a specialized tumor board. Eligibility is confirmed through diagnostic tests according to local criteria, and the patient is subsequently referred to an authorized CAR T-cell center. As discussed previously, depending on the country, formal approval is required by payers and regional and/or national authorities before CAR T-cell therapy. For CAR T-cell production, the patient must undergo leukapheresis, usually at the authorized center, where T-cells are collected and shipped to a production facility of the pharmaceutical manufacturer. Before infusion of the CAR T-cells by the authorized center, patient will receive bridging therapy for disease control if required, and lymphodepleting conditioning chemotherapy to create an optimal environment for CAR T-cells expansion after infusion. After CAR T-cell infusion, guidelines recommend that the patient remains at the CAR T-cell center for 10 to 14 days, and in the vicinity of the center until day 28 post-infusion to ensure appropriate monitoring of adverse events. Usually, the patient is referred back to their referring physician for long-term follow-up and care (2, 3, 36).

Owing to the innovative nature and complexity of CAR T-cell therapy, implementation into routine care has proven challenging for health systems. This publication analyzes DLBCL patients’ access to licensed CAR T-cell therapies in the four largest EU countries and discusses the main challenges for patient access and recommends solutions for overcoming these challenges for current and future CAR T-cell therapies.

## 2. DLBCL CAR T-cell therapy access situation in EU-4 countries in 2020: analysis of unpublished data

To assess the CAR T-cell therapy access situation for DLBCL patients across the EU-4 countries, we compared the EMA label population (R/R DLBCL patients after at least two therapy lines) as well as the estimated fraction that is considered medically eligible for CAR T-cell therapy, with the number of R/R DLBCL patients treated with licensed CAR T-cell therapies in the year 2020. To ensure comparability of the analysis across the EU-4 countries, a normalization by the respective population size was conducted (Supplementary Table 2). The size of the EMA DLBCL label population was derived or estimated based on publicly available data (Supplementary Table 2). The fraction of CAR T-cell therapy eligible patients was estimated based on the registrational trial information for *tisagenlecleucel* and *axicabtagene ciloleucel*, the same approach as used by HAS (French National Authority for Health) to estimate the number of potential CAR-T patients in France (using the average percentage of patients selected for CAR T-cell therapy that in the end received treatment; Supplementary Table 2). No publicly available data could be identified reporting the number of R/R DLBCL patients treated with licensed CAR T-cell therapies for the full year 2020 (January to December) in the EU-4 countries. Published registry data from France (DESCAR-T), Germany (DRST), Italy (AIFA) and Spain (VALTERMED) cover different timeframes, aggregating patients treated since registry opening, and may not be complete (26, 28, 37, 38). To overcome this limitation, our analysis used data and estimations for the total number of licensed CAR T-cell therapies (*tisagenlecleucel* and *axicabtagene ciloleucel*) for R/R DLBCL patients in France, Germany, and Italy in the year 2020, that were provided in personal communication by Kite Pharma Inc./ Gilead Sciences Inc. (39). For Spain, in response to a request for data disclosure, the Ministry of Health reported the number of R/R DLBCL patients for whom a licensed CAR T-cell therapy was requested in 2020, as well as the number of approved requests and the number of performed CAR T-cell therapies for these patients (40).

**Figure 1:**
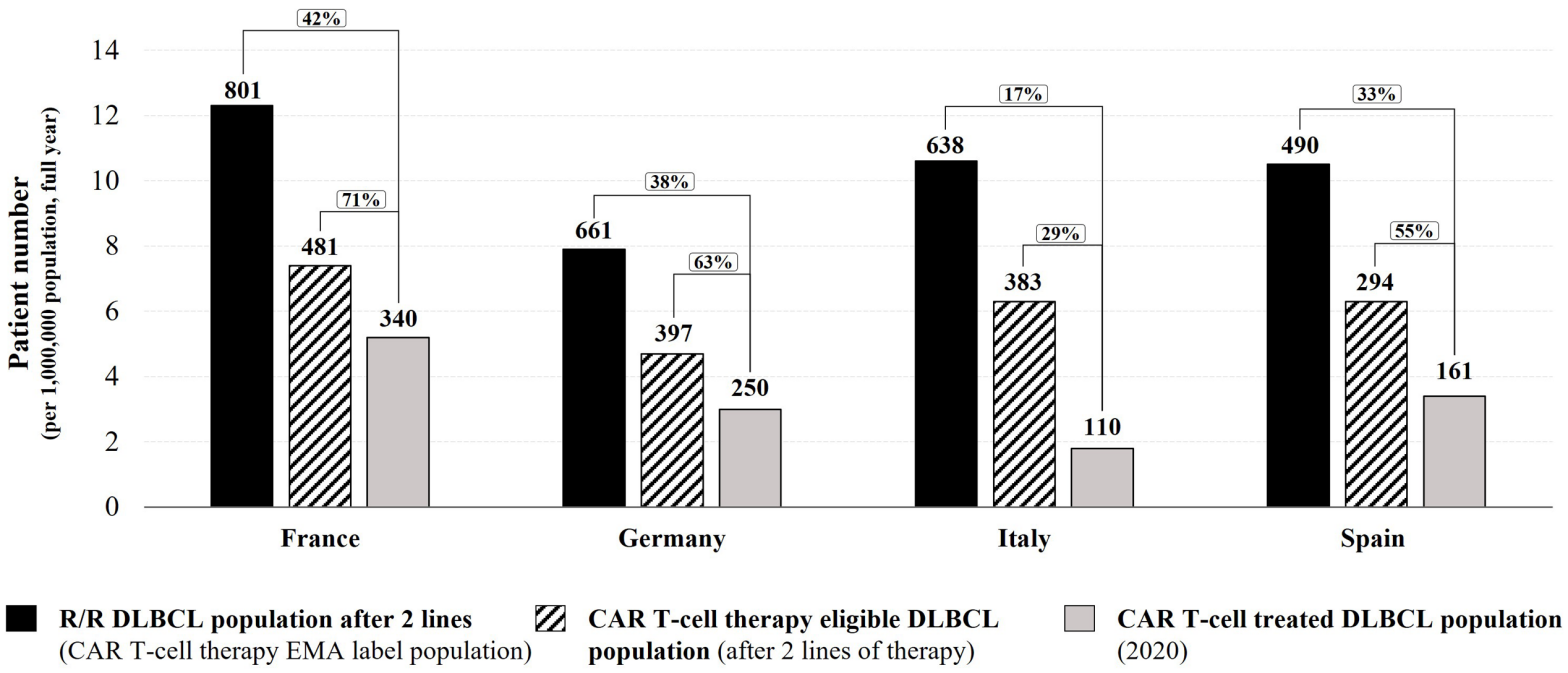
2020 DLBCL CAR T-cell therapy access analysis in Germany, France, Italy, and Spain. Numbers in graph indicate total patient numbers in the EU-4 countries. Bar graph height represents patient numbers normalized per 1 million inhabitants (relative to country population size in 2020). R/R – relapsed/refractory.

We analyzed patient access to CAR T-cell therapies across the ‘EU-4’ countries (France, Germany, Italy, and Spain), comparing the EMA DLBCL label population (R/R DLBCL patients after at least two lines of therapy) as well as the fraction estimated to be medically eligible with the number of R/R DLBCL patients effectively treated with a licensed CAR T-cell therapy in the year 2020 (Figure 1; Supplementary Table 2).

Based on public information and our calculations for the year 2020, the numbers of DLBCL patients relapsed/refractory after at least two therapy lines (corresponding to the EMA label population for DLBCL CAR T-cell therapies) were: 801 in France (12.3 per one million in habitants), 661 in Germany (7.9 per one million inhabitants), 638 in Italy (10.6 per one million in habitants), and 490 in Spain (10.5 per one million inhabitants) (Figure 1; Supplementary Table 2). However, not all R/R DLBCL patients with two or more lines of therapy are considered medically eligible for CAR T-cell therapy due to their general health and disease status. Based on the registrational trial information for *tisagenlecleucel* and *axicabtagene ciloleucel*, on average around 60% of selected patients had been treated in registrational trials. Therefore, we estimated that for the year 2020, 481 DLBCL patients in France would have been considered medically eligible to undergo licensed CAR T-cell therapy, 397 patients in Germany, 383 patients in Italy, and 294 patients in Spain [Figure 1; Supplementary Table 2; (17, 18)]. Based on information available for 2020, 340 DLBCL patients in France received a licensed CAR T-cell therapy [28 patients per month (39)], 250 DLBCL patients in Germany [21 patients per month (39)], 110 DLBCL patients in Italy [9 patients per month (39)], and 161 DLBCL patients in Spain [13 patients per month (40); Figure 1]. The above estimations for France and Italy are in range of DLBCL CAR T-cell therapy rates reported by the DESCAR-T registry (30 patients per month on average between December 2019 to January 2021) and the AIFA registry (8 patients per month on average between August 2019 and December 2020) (24, 28). For Germany, the DRST registry data from participating CAR T-cell centers suggests a lower monthly DLBCL CAR T-cell therapy rate than estimated by our analysis (15 patients per month on average between November 2018 and April 2021, after extrapolation to all 29 active centers in December 2020). This difference might be due to the published DRST registry data including a long time period (from November 2018) during which CAR T-cell therapy might have been used only by a few centers and clinical practice was slowly building up [Supplementary Table 2; (24, 26, 28, 39)].

This analysis indicates that in all four EU countries, less than 50% of DLBCL patients matching the EMA label indication received a CAR T-cell therapy in 2020 (Figure 1). Of the estimated CAR T-cell therapy eligible patients, approximately 7 in 10 DLBCL patients in France have been treated with a licensed CAR T-cell therapy in 2020 (71%; 42% of the EMA label population). In Germany and Spain, the CAR T-cell therapy rate was approximately 6 in 10 eligible DLBCL patients (63% and 55% respectively, 38% and 33% of the EMA label population), and in Italy, less than 3 in 10 eligible DLBCL patients received a CAR T-cell therapy in 2020 (29%, 17% of the EMA label population). It should be noted that despite a larger population size in Germany (83.8 million), the German Federal Joint Committee G-BA’s averaged estimations for the number of R/R DLBCL patients after at least two lines of therapy (661) is in range with estimations for Italy (638 patients in 60.5 million inhabitants) and is lower than estimations for France [801 patients in 65.3 million inhabitants; Figure 1; Supplementary Table 2; (12, 17, 18, 20, 41–44)]. As emphasized by the German HTA institute IQWiG in its assessment report, the estimation of R/R DLBCL patients after two lines of therapy in Germany might be underestimated due to data and methodical uncertainties (41); consequently, the rate of DLBCL CAR T-cell therapies in Germany in 2020 might be lower than reported in this analysis. In summary, between 29 and 71% of the estimated medically eligible patients did not receive a licensed CAR T-cell therapy in 2020, despite promising long-term efficacy and survival data (45–47). Reasons for this shortfall might include use of alternative therapies or clinical trial enrollment, restrictive funding criteria applied (e.g., CAR T-cell therapy not funded for DLBCL patients above a maximum age of 70 in Italy (increased to 75 years in May 2022) (23–25).

The number of authorized centers per population might also influence the overall CAR T-cell therapy rate. At the end of 2020, France, Germany, and Italy had a similar density of active DLBCL CAR T-cell centers (one center for two to three million inhabitants), whereas in Spain, center density was almost half of that (one center for five million inhabitants; Supplementary Figure 1; Supplementary Table 3). Of the EU-4 countries, Spain had the highest utilization of its DLBCL CAR T-cell centers as measured by the average number of DLBCL patients treated with licensed CAR T-cell therapies per center in 2020 (18 patients per center), followed by France (14 patients per center), Germany (9 patients per center) and Italy (6 patients per center; Supplementary Figure 1; Supplementary Table 3), indicating that Spain could partially compensate for its lower number of centers through a higher average treatment rate per center, although still not achieving overall CAR T-cell therapy rates at the level of France or Germany. Conversely, despite having a similar center density as France or Germany, average utilization of DLBCL CAR T-cell centers in Italy was the lowest, correlating with the lower rate of licensed CAR T-cell therapies overall. Unfortunately, no information on the theoretical CAR T-cell therapy capacity per center could be identified.

The following chapter will discuss challenges faced by DLBCL patients along their patient journey, which might contribute to the differences in CAR T-cell therapy rates observed among the EU-4 countries.

## 3. Challenges, best practices, and health system focus areas for DLBCL CAR T-cell therapy access in EU-4 countries: review of the literature and experts’ opinion

To identify and discuss challenges, existing best practices, and potential health system recommendations for CAR T-cell therapy access in the EU-4 countries, the authors’ professional experience, expertise and personal views were recorded through individual phone interviews (Supplementary Table 4 with interview questions). The authors have been selected based on their relevant expertise with CAR T-cell therapies in the four largest EU countries [convenience sample (48)] and the findings included in this chapter represent their personal viewpoints and experiences, substantiated by literature review.

To capture the challenges that might limit patient access to licensed CAR T-cell therapies and the differences seen across the EU-4 countries, we analyzed the key steps along the DLBCL patient journey, namely patient identification and referral (chapter “Patient identification and referral for CAR T-cell therapy”), patient approval before treatment (chapter “Patient approval before CAR T-cell therapy”), and CAR T-cell therapy delivery at authorized centers (chapter “CAR T-cell therapy delivery at authorized centers”). In addition to the identified challenges, we also discuss best practices and potential health system focus areas for improving patient access to CAR T-cell therapies.

### 3.1. Patient identification and referral for CAR T-cell therapy

#### 3.1.1. Challenges for patient identification and referral

DLBCL is a fast-progressing disease, particularly in refractory patients, with a median overall survival of 6.3 months after start of second line therapy (33). Accurate and timely identification and referral of eligible R/R DLBCL patients for CAR T-cell therapy are therefore essential to ensure optimal outcomes and might represent a significant challenge for access to CAR T-cell therapies. Based on data available for Spain in 2020, for an estimated 294 medically eligible R/R DLBCL patients only 200 (68%) requests for licensed CAR T-cell therapy were submitted to the national expert group (40, 49), suggesting that up to one in three eligible patients were not identified or referred for CAR T-cell therapy.

Patient identification and selection for CAR T-cell therapy can be particularly challenging in small peripheral hospitals and clinics that treat many types of malignancies and may have only limited specific knowledge of the benefits and eligibility criteria of CAR T-cell therapies. Moreover, peripheral hospitals may not always be strongly connected with CAR T-cell centers or integrated with hemato-oncological care networks, where existing, which could support patient identification and referral for CAR T-cell therapies. The absence of clear and well-defined referral pathways for CAR T-cell therapies may also limit the likelihood and timeliness of referral of an eligible DLBCL patient to a CAR T-cell center. In addition, treating hematologists might be hesitant to refer eligible patients due to the perceived complexity and duration of the CAR T-cell therapy process, potentially prioritizing local treatment options, which further delay patient access to a CAR T-cell therapy. Despite being indicated after two lines of therapy, data from different European registries shows that many DLBCL patients treated or approved for treatment with CAR T-cell therapies had received three or more lines of prior therapies [71% of patients with at least three lines of therapy in the German DRST registry (26), 50% of patients in the French DESCAR-T registry (28), 42% of patients in the Italian AIFA registry (37), and 39% of patients in the UK national program (50)]. These findings suggest that referral of these patients potentially occurred too late and that timely identification and referral is a shared challenge across major European countries. Of note, it is likely that a number of patients included in these registries had already received several lines of therapy before CAR T-cell therapies became locally approved, and therefore might have been overrepresented at the time of registry opening.

CAR T-cell center density per population (discussed above; Supplementary Figure 1; Supplementary Table 3) and moreover uneven geographical distribution of centers, may also have a role in creating referral delays or hesitancy, particularly if patients are required to travel over large distances to undergo CAR T-cell therapy. In Germany, personal experience from the authors suggests that travel distances of up to 2 hours to receive post-therapy follow-up or adverse event management create significant challenges, particularly for elderly patients. In Spain, where authorities designated nine CAR T-cell centers (and two “back-up” centers in case of capacity need), only six out of 17 regions had an active DLBCL CAR T-cell center by March 2021 (38). Out-of-region referral in Spain was reported to delay CAR T-cell therapy by 6 days compared to patients who have a CAR T-cell center in their region (67 days vs. 61 days median duration from treatment approval request to infusion, data from March 2019 – March 2021, including DLBCL and pediatric ALL CAR T-cell therapies) (22, 38). To ensure broader access to CAR T-cell therapy for lymphoma and leukemia patients across the country, the Spanish Ministry of Health (SNS) designated 15 new CAR T-cell centers in June 2022, including several in regions that did not have a CAR T center so far (51). In Italy, initial delays of center authorization and qualification by regional authorities and pharmaceutical manufacturers resulted in a geographically heterogeneous coverage of CAR T-cell centers. In December 2020, 70% of active CAR T-cell centers were concentrated in only four Italian regions (37). Regional inequities appear to have affected patient access in Italy. From August 2019–December 2020, DLBCL patients undergoing CAR T-cell therapies came from only 10 out of 20 regions (37).

#### 3.1.2. Best practices and health system focus areas for patient identification and referral

Examples of successfully implemented best practices to address patient identification and referral challenges for CAR T-cell therapies are discussed in the following.

##### 3.1.2.1. Educational activities

National and regional workshops and virtual roadshow events between CAR T-cell and referring centers provide an opportunity to discuss the CAR T-cell therapy process and patient eligibility criteria with referring physicians. Such meetings, as implemented by many European CAR T-cell centers, also support exchange of learnings and best practices for patient selection, such as for complex cases with specific comorbidities, reduced health status or age.

##### 3.1.2.2. CAR T-cell therapy referral networks and quality criteria

To support early patient identification and timely referral, the hematology network around the Centre Hospitalier Universitaire de Montpellier has expanded its regional multi-disciplinary committee meeting to also include a CAR T-cell therapy dedicated segment. In the weekly virtual meeting, the committee reviews all presented DLBCL patients in the southern French region Occitania and advises referring physicians on CAR T-cell therapy eligibility and a course of treatment as soon as a patient fails first line therapy. The regional hematology network also defined clear pathways and quality criteria for the CAR T-cell therapy referral process (for instance final confirmation of patient eligibility must happen not more than 8 days after discussion in the regional committee, with clear responsibilities for diagnosis at both referring and CAR T-cell center). Similar network structures and regional dedicated multi-disciplinary meetings for CAR T-cell therapies exist also in other French regions (for instance implemented by the Hospices Civils de Lyon) and their development is supported by the French authorities through the National Cancer Plan (52). In the German region Rhineland-Palatinate, CAR T-cell centers and larger hospitals providing second-line therapy collaborate in a competence network for stem cell transplantation and cellular therapy, developing CAR T-cell therapy criteria and pathways, and organizing weekly virtual meetings to discuss individual patient cases. Outpatient hematology care providers are, at time of writing, not yet included in the network. In Spain, virtual weekly CAR T-cell committee meetings, now established in the Madrid region for example, also support referring centers with identification and referral of eligible patients. Beyond that, the Catalunya region has also established a well-defined hematology network with clear referral pathways that is also being used for CAR T-cell therapy patients. Small peripheral hospitals are strongly connected with larger hospitals, which in turn each have a designated reference CAR T-cell center where potential patients are referred to. The regional network also ensures continuous education of regional hospitals, ensuring that CAR T-cell therapy benefits, safety aspects and selection criteria are well known.

Based on the above discussed challenges and best practice examples, the authors suggest four key areas of focus for health systems to overcome challenges with patient identification and referral for CAR T-cell therapies (Table 2).

**Table 2:** Key health system focus areas for patient identification and referral challenges.

A. Focused referring center education on CAR T-cell therapy benefits/risks, patient selection and referral process, by CAR T-cell centers and hematology networks
B. Strengthened hematology networks, integrating referring centers through (virtual) tumor boards with a CAR T-dedicated segment and active communication among all network participants, including health authorities
C. Clear pathways, quality standards and responsibilities for patient referral for CAR T-cell therapy
D. Optimized geographical distribution of CAR T-cell centers to ensure equal access and minimal travel burden for patients

### 3.2. Patient approval before CAR T-cell therapy

#### 3.2.1. Challenges

As discussed before, funding approval before CAR T-cell therapy is required in Spain (approval by the region of patient origin and by the national CAR T-cell therapy expert group). Depending on the center, pre-treatment funding approval may be required in Germany (approval by the patient’s sick fund and medical review board), and in Italy (approval by the regional authorities). Without guarantee that the CAR T-cell product and procedure costs are funded, it is unlikely that authorized centers will initiate the treatment due to the high financial risks.

Collecting funding approval before CAR T-cell therapy can be a long and complex multi-step process adding administrative burden at the authorized centers and potentially resulting in delays in CAR T-cell therapy access for patients. Registry data from patients treated in Germany and Spain report a median duration of 26 and 17 days respectively, from the moment of clinical decision for CAR T-cell therapy until leukapheresis, which besides other aspects also reflects the time required to receive external funding approval [Spanish data including DLBCL and also pediatric ALL patients; (26, 38)]. For patients whose health deteriorates during this wait time so much that they cannot undergo a CAR T-cell therapy in the end, such delays effectively restrict access to this therapy and its potential benefits. In Germany, personal experience from the authors indicates that it can take up to 4 weeks to receive funding approval by certain sick funds. These long timelines are in part also due to paper-based communication and the absence of an electronic information-sharing system. In addition, as no nationally harmonized criteria for CAR T-cell therapy eligibility are published and applied in Germany, decision making on patient eligibility can differ across sick funds, increasing financial and process uncertainties for CAR T-cell centers. In Spain, decisions for CAR T-cell therapy eligibility by the national expert group have occurred within 24 hours in 68% of “vital urgent cases” and within 72 hours in 79% of “non-vital urgent cases,” however in a minority of cases the decisions took more time [data for March 2019 to March 2021 (38)]. Additional delays are thought to occur from the funding approval step with regional health authorities in Spain. For instance, based on author experience, decisions by authorities in the Catalunya region usually take around 2–3 days. Conversely, no additional delays are thought to occur in the Madrid region as the regional health authorities in Madrid only centralize and process requests for CAR T-cell therapy to the national expert group. In France and Italy, national financing mechanisms for innovative medicines are available for CAR T-cell therapies [“*Liste en Sus*” and “*Fondo Farmaci Oncologici Innovativi*” (29)] under the condition that information is provided through a national registry to authorities. This results in funding certainty for authorized centers and absence of a pre-treatment funding approval step in most cases. However, authorized private CAR T-cell centers in Italy cannot directly receive the national innovation funding, and instead require pre-treatment approval and CAR T-cell product purchase by the regional authorities or a public hospital in their place. In addition, for patients referred from other regions, to ensure that procedure costs are covered, authority approval from the region of patient origin may also be required in certain regions before initiation of therapy. This has resulted in delay in patient access to CAR T-cell therapies in Italy.

#### 3.2.2. Best practices and health system focus areas

Examples of successfully implemented best practices to address patient approval challenges for CAR T-cell therapies are discussed in the following.

##### 3.2.2.1. CAR T-cell therapy decision making by authorized centers

In France, authorized CAR T-cell centers decide on patient eligibility based on their medical assessment and in compliance with the licensed EMA label indication, but independent of an authority approval requirement. This approach has also allowed authorized centers to continuously refine patient selection based on their experiences and new evidence becoming available, effectively ensuring that all DLBCL patients that could benefit from CAR T-cell therapies have timely access to treatment. In Germany, most CAR T-cell centers have developed internal checklists over time based on their experience with the local sick fund process that allow them to anticipate specific requirements for eligibility decision making and to reduce the overall duration of pre-treatment funding approval.

##### 3.2.2.2. National CAR T-cell therapy eligibility criteria and harmonized approval process

In Italy and Spain, the published and nationally harmonized DLBCL CAR T-cell therapy eligibility criteria, while restricting the eligible population beyond the EMA label, ensure transparency for patients and healthcare professionals and allow uniform decision-making on patient access to CAR T-cell therapies (23, 24, 38). In Germany, with the aim to work toward harmonization of patient eligibility criteria and decision-making, a monthly conference between the national Competence Center Oncology KCO, the sick fund’s medical review boards and CAR T-cell centers, was initiated in 2021 (53). Centralized approval processes can also support ensuring equal access to CAR T-cell therapies for DLBCL patients. In the UK, a weekly National CAR T Clinical Panel (NCCP), composed of clinical experts, patient representatives and CAR T-cell center delegates decides on patient eligibility, prioritization, and referral to an appropriate center depending on available capacity and geographic vicinity (49, 54). A national CAR T-cell therapy board supporting efficient decision making on patient eligibility and referral was also established in the Netherlands by the hemato-oncology society HOVON (55). Similar to the centralized approval processes in the UK and the Netherlands, the Spanish national approval process aims to ensure equal patient access but is linked to prior regional authority approval, which may delay the process and potentially result in regionally heterogeneous decision-making. Of note, all described national approval processes may carry the risk of becoming bottlenecks to patient access in the future, when more patients in new indications are expected to become eligible for CAR T-cell therapy.

Based on the above discussed challenges and best practice examples the authors suggest three key areas of focus for health systems to overcome challenges with patient approval before CAR T-cell therapies (Table 3).

**Table 3:** Key health system focus areas for patient approval challenges.

A. Ensuring nationally harmonized and transparent CAR T-cell therapy eligibility criteria to support equal decision making and clarity on patient selection
B. Simplification of pre-treatment approval process through digitalization and reduction of number of process steps (e.g., single-step approval process, either at the regional or national level)
C. Decision making directly by authorized CAR T-cell centers without regional/ national authority or payer pre-treatment approval requirement (for instance, with control through a registry documentation requirement)

### 3.3. CAR T-cell therapy delivery at authorized centers

#### 3.3.1. Challenges

CAR T-cell centers require specific capabilities, organization, and infrastructure to ensure optimal and timely delivery of CAR T-cell therapy to eligible patients. Specifically trained interdisciplinary teams are involved through various processes required for CAR T-cell therapy administration and patient management. Due to the complexity of CAR T-cell therapies, authorized centers may face challenges in providing CAR T-cell therapy capacity in a short time window to address the urgent medical need of R/R DLBCL patients, and in avoiding delays to other patient treatments that depend on similar center resources. With more patients expected to undergo CAR T-cell therapies in future indications, capacity challenges at authorized centers are likely to increase further.

Limited center resources for CAR T-cell therapy delivery may arise from lack of support from authorities to ensure financing for infrastructural and organizational investments required for CAR T-cell therapy. For instance, centers needed to invest in expanding their hematology wards and ICU beds, as CAR T-cell therapy patients were required to stay in the center for up to 3 weeks after infusions (2, 3, 26, 36, 38). Also, leukapheresis, cell therapy laboratory and pharmacy capacity needed to be increased, which proved to be difficult for certain CAR T-cell centers based on the authors’ experience. Without financial support from authorities, such investments had to be covered by the centers independently, potentially resulting in delays with center authorization or limited overall CAR T-cell therapy capacity. In addition to challenges with infrastructural investments, the build-up of highly specialized teams, especially nurses, physicians, and pharmacists, requires significant training investment and may become increasingly difficult. CAR T-cell center quality criteria for instance, as introduced by the German Federal Joint Committee G-BA, include strict requirements on the professional qualifications of CAR T-cell therapy nurses (20), making it increasingly challenging to identify and recruit appropriately qualified nurses. Non-competitive compensation in public hospitals may increase staffing challenges further.

Moreover, beyond costs of the CAR T-cell product, centers must also cover costs of the procedure, including provision of specific care, monitoring, and hospital resources. Such costs might not be covered adequately through existing funding, resulting in additional financial challenges for authorized centers. In France for instance, a study reported that unlike CAR T-cell product costs, procedure costs were covered through a general, non-specific tariff resulting in an approximate €19,000 loss per DLBCL patient treated with CAR T-cell therapy (56). In Italy there are not CAR-T cell specific tariffs for the relevant inpatient service, which are classified according to the DRG (Diagnostic-Related-Groups) system. Currently, regions use different tariffs to cover CAR T-cell therapy procedures, for instance tariffs for allogeneic or autologous stem cell transplantation. There is no evidence whether these tariffs cover the relevant procedure costs (57). In Germany, hospitals may ask for funds for innovative and complex medicines/procedures through the NUB. Both CAR-T cell received a NUB 1 rank, which is granted to a medicine or procedure which is new, innovative, for a low number of patients and which requires higher resource (58). In Spain, the costs of the drug are covered by the patient’s local hospital, whereas the cost of care and procedures are covered by the Autonomous Community where the treatment takes place, and subsequently refunded via the Health Cohesion Fund (30, 59, 60). In France, hospitals receive a flat supplement of 15,000 € per patient to cover the procedures associated with the CAR T treatment (61).

#### 3.3.2. Best practices and health system focus areas

Examples of successfully implemented best practices to ensure adequate capabilities, organization, and infrastructure for CAR T-cell therapies are discussed in the following.

##### 3.3.2.1. Constructive collaboration and support from health authorities

In France, CAR T-cell therapies were introduced in specialized centers with direct support from the regional health authorities, through collaborations that initiated already before EMA regulatory approval in the context of the ATU early access program (17, 18). Under the National Cancer Plan this collaboration between centers and authorities is expected to continue also for new CAR T-cell therapy indications and future capacity expansions required.

##### 3.3.2.2. Optimizing CAR T-cell therapy capacity by utilizing network resources and reducing inpatient care

To optimize CAR T-cell therapy capacity and resource use, an approach taken by certain authorized centers is to delegate selected CAR T-cell therapy process steps within their network. In Spain, leukapheresis for CAR T-cell production is also provided at the level of certain specialized referring centers. Besides improving available capacity at the CAR T-cell center, this approach also reduces travel requirements for patients undergoing CAR T-cell therapy. Similarly, in France collaboration with the French Blood Collection Association EFS allowed to increase leukapheresis capacity outside of CAR T-cell centers. Furthermore, specialized rehabilitation centers for follow-up monitoring and accommodation after CAR T-cell infusion are being used to optimize bed capacity in CAR T-cell centers in Montpellier and Lyon. Moreover, reducing the requirement for inpatient procedures might also improve overall efficiency of resource usage. By conducting lymphodepletion in an outpatient setting, the Centre Hospitalier Universitaire de Montpellier was able to reduce hospitalization per patient by 3 days, effectively increasing their overall CAR T-cell therapy capacity. Other evidence suggests that in the future more procedures of CAR T-cell therapy may be provided in an outpatient setting (62).

Based on the above discussed challenges and best practice examples the authors suggest three key areas of focus for health systems to ensure adequate set-up of authorized centers for CAR T-cell therapies (Table 4).

**Table 4:** Key health system focus areas for ensuring adequate CAR T-cell center set-up.

A. Early and adequate authority support and coordinated capacity planning for CAR T-cell centers, also supporting professional training for specialized personnel
B. Leveraging network resources to optimize overall CAR T-cell therapy capacity (e.g., leukapheresis and post-infusion care performed in referring centers). Capacity optimization should also occur across networks, where patients are referred from one CAR T-cell center to another in case of limited treatment capacity
C. Sufficient CAR T-cell therapy procedure funding for authorized centers, ideally through a dedicated national tariff

## 4. Discussion

CAR T-cell therapies offer a promising new therapeutic approach to treating a number of severe cancers, including DLBCL where R/R patients previously had only very limited therapeutic options (33). Despite promising results of CAR T-cell therapies (45–47), our analysis indicates that between 29% and 71% of the estimated eligible R/R DLBCL patients have not been treated with a licensed CAR T-cell therapy in 2020 in the EU-4 countries. While reasons for a low DLBCL CAR T-cell therapy rate can be diverse, including rapid disease progression and worsening of the patient’s condition before treatment, use of alternative therapies or clinical trial enrollment, our analysis highlights a critical role for the health system in optimizing the patient’s journey and access to CAR T-cell therapies. DLBCL CAR T-cell therapy rates were highest in France (71%). Reasons for this may include the early introduction of CAR T-cell therapy through the early access program ATU, allowing for early accumulation of expertise before EMA approval, the strongly embedded regional hematology networks supporting the CAR T-cell therapy patient journey, the national CAR T-cell therapy funding through the “*Liste en Sus*,” as well as authority support driven by the National Cancer Plan (17, 18, 29, 52). Conversely in Italy, DLBCL CAR T-cell therapy rates were the lowest (29% of the estimated eligible patients) among the EU-4 countries in 2020 based on our analysis. Reasons for this may include the restrictive funding criteria for R/R DLBCL patients (including a maximum age for patient eligibility) and a lack of harmonized processes for managing CAR T-cell therapy pathways in a regionalized health care system, which could particularly result in challenges with timely patient identification and referral. The AIFA registry suggests that regional inequalities exist in patient access to CAR T-cell therapies, potentially also linked to a concentration of CAR T-cell centers in few Italian regions (37).

It must be noted that year 2020 has posed special challenges on the healthcare systems due to the COVID-19 pandemic, which affected all healthcare provisions, including specialized therapies like CAR T cell therapy. However, the authors are convinced that most of the barriers discussed in this manuscript are of systemic nature and not directly resulting from the pandemic situation, although they were likely further aggravated. Furthermore, the paper has carried out a cross-country comparison, and all analyzed countries have been affected by COVID-19 pandemic.

It should be noted that as we report data on the CAR T-cell therapy rate in 2020, the general patient access situation has likely evolved in the meantime, with additional expertise having been gained and additional CAR T-cell centers having been authorized. More research and systemic data collection will be needed to understand CAR T-cell therapy pathways and challenges along the patient journey in further detail. For instance, registries as implemented for CAR T-cell therapies in the EU-4 countries represent an important tool for tracking and improving health system performance for CAR T-cell therapy patients. However, these registries require further expansion (e.g., the inclusion of referral rates and timelines for CAR T-cell therapies) and need to provide a more systematic read out to support health system planners and decision-makers with the necessary information to focus improvement efforts.

## 5. Conclusion

Our analysis across the four largest health systems in the European Union has identified several challenges that can impact timely and equitable access to CAR T-cell therapy for eligible DLBCL patients. With additional CAR T-cell therapies for hematological cancers entering the market in the coming years (and potential future CAR T-cell treatments for solid tumors), we believe that it is crucial to ensure that health systems act on these challenges now and work to prepare a sustainable environment that will support patient access to future innovative therapies. The best practices and focus areas discussed in this article can serve as a blueprint to initiate improvements designed to fit within their national and local health system environments.

## Data availability statement

The original contributions presented in the study are included in the article/Supplementary material, further inquiries can be directed to the corresponding author.

## Author contributions

MÁCA, PLC, GC, JC, BD, CJ, RM, CR, AS, JMSC, and EMW-D contributed to the analysis of the CAR T-cell therapy access situation, barriers, and best practices, to the discussion of health system recommendations and to the writing of the manuscript. All authors contributed to the article and approved the submitted version.

## Funding

Funding for journal publication fees and manuscript writing support by the agency Executive Insight was provided by Kite Pharma Inc./ Gilead Sciences Inc. Kite Pharma Inc./ Gilead Sciences Inc. contributed to the selection of authors but had no direct involvement in the preparation and development of the publication.

## Conflict of interest

The authors declare that the research was conducted in the absence of any commercial or financial relationships that could be construed as a potential conflict of interest.

## Publisher’s note

All claims expressed in this article are solely those of the authors and do not necessarily represent those of their affiliated organizations, or those of the publisher, the editors and the reviewers. Any product that may be evaluated in this article, or claim that may be made by its manufacturer, is not guaranteed or endorsed by the publisher.
