# Supplementary material for: Comparative analysis of CAR T-cell therapy access for DLBCL patients: associated challenges and solutions in the four largest EU countries
Source: Front Med (Lausanne). 2023 May 30;10:1128295. doi: 10.3389/fmed.2023.1128295 (PMC10263061; doi:10.3389/fmed.2023.1128295)
Supplement: Supplementary file 1 [file Data_Sheet_1.docx]

***Supplementary Material***

**Supplementary Figure 1.** 2020 DLBCL CAR T-cell center density and utilization in France, Germany, Italy, and Spain.

*Numbers in graph indicate total DLBCL CAR T-cell center numbers in the EU-4 country in 2020. Bar graph height is relative to DLBCL CAR T-cell centers normalized by the countries’ relative population size in 2020 (per one million inhabitants). Points on dotted line indicate average number of CAR T infused DLBCL patients per DLBCL CAR T-cell center in 2020. References and calculations in Supplementary Table 3.*


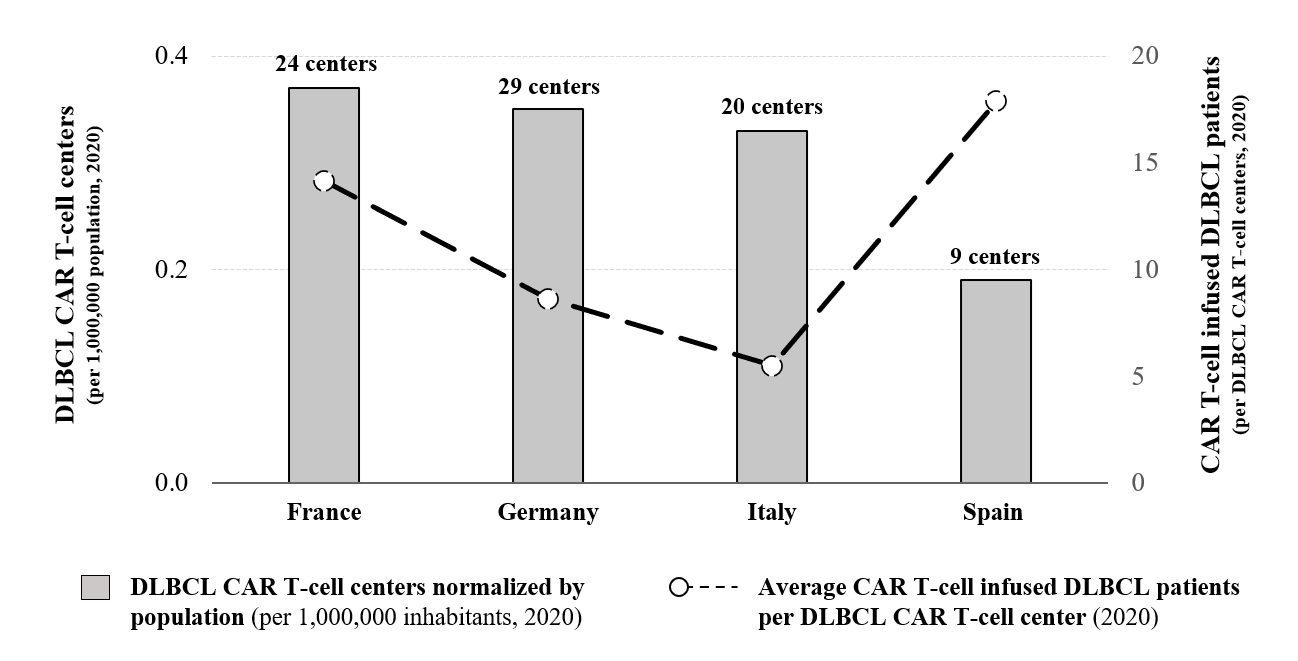


**Supplementary Table 1.** Indications and commercial CAR T-cell therapies approved by the European Medicines Agency (EMA) and the U.S. Food and Drug Administration (FDA) (May 2022)

| **Indication** | **Target antigen** | **Commercial CAR T-cell therapy** | **Date of EMA approval** | **Date of FDA approval** |
| --- | --- | --- | --- | --- |
| Adult R/R DLBCL  For patients after two or more lines of systemic therapy (EMA/FDA).*  For patients refractory to first line chemoimmunotherapy or relapsed within 12 months of first line chemoimmunotherapy (FDA).** | CD19 | **tisagenlecleucel* | September 2018 (2) | May 2018 (63) |
|  |  | **/**axicabtagene ciloleucel* | September 2018 (3) | *October 2017 (64)  **April 2022 (64) |
|  |  | **lisocabtagene maraleucel* | April 2022 (4) | February 2021 (65) |
| Adult R/R PMBCL  For patients after two or more lines of systemic therapy (EMA/FDA). | CD19 | *axicabtagene ciloleucel* | September 2018 (3) | October 2017 (64) |
|  |  | *lisocabtagene maraleucel* | April 2022 (4) | February 2021 (65) |
| Adult R/R HGBL  For patients after two or more lines of systemic therapy (FDA). | CD19 | *tisagenlecleucel* | - | May 2018 (63) |
|  |  | *axicabtagene ciloleucel* | - | October 2017 (64) |
|  |  | *lisocabtagene maraleucel* | - | February 2021 (65) |
| Adult R/R DLBCL arising from FL  For patients after two or more lines of systemic therapy (FDA). | CD19 | *tisagenlecleucel* | - | May 2018 (63) |
|  |  | *axicabtagene ciloleucel* | - | October 2017 (64) |
|  |  | *lisocabtagene maraleucel* | - | February 2021 (65) |
| Adult R/R FL  For patients after two or more lines of systemic therapy (EMA).*  For patients after three or more lines of systemic therapy (EMA).**  For patients after two or more lines of systemic therapy (FDA).**  For patients with grade 3B FL after two or more lines of systemic therapy (EMA/FDA).*** | CD19 | **tisagenlecleucel* | April 2022 (2) |  |
|  |  | ***axicabtagene ciloleucel* | April 2022 / CHMP positive opinion (6) | April 2021 (64) |
|  |  | ****lisocabtagene maraleucel* | April 2022 (4) | February 2021 (65) |
| Pediatric R/R ALL  For patients up to 25 years who are refractory, or in second or later relapse (EMA/FDA).  For patients up to 25 years who are in relapse post-transplant (EMA). | CD19 | *tisagenlecleucel* | September 2018 (2) | August 2017 (63) |
| Adult R/R ALL  For patients after one or more lines of systemic therapy (FDA). |  | *brexucabtagene autoleucel* |  | October 2021 (66) |
| Adult R/R MCL  For patients after two or more lines of systemic therapy including a BTK inhibitor (EMA).  For patients after one or more lines of systemic therapy (FDA). | CD19 | *brexucabtagene autoleucel* | December 2020 (7) | July 2020 (66) |
| Adult R/R MM  For patients after three or more lines of systemic therapies, including an immunomodulatory agent, a proteasome inhibitor and an anti CD38 antibody, and that have demonstrated disease progression on the last therapy (EMA).  For patient after four or more lines of systemic therapy including an immunomodulatory agent, a proteasome inhibitor, and an anti-CD38 monoclonal antibody (FDA). | BCMA | *idecabtagene vicleucel* | August 2021 (8) | March 2021 (8) |
|  |  | *ciltacabtagene autoleucel* | March 2022 / CHMP positive opinion (9) | February 2022 (9) |

*ALL - B-cell precursor acute lymphoblastic leukemia, BCMA - B-cell maturation antigen, BTK - Bruton’s tyrosine kinase, CD19 - B-lymphocyte antigen CD19 (Cluster of Differentiation 19), DLBCL - Diffuse large B-cell lymphoma, FL - Follicular lymphoma, HGBL – High-grade B-cell lymphoma, MCL - Mantle cell lymphoma, MM - Multiple myeloma, PMBCL - Primary mediastinal large B-cell lymphoma.*

**Supplementary Table 2.** 2020 DLBCL CAR T-cell therapy access analysis in France, Germany, Italy, and Spain.

|  | **Absolute numbers** | | | | **Normalized by population**  (rate per one million) | | | | **Comments / References** | | | | |
| --- | --- | --- | --- | --- | --- | --- | --- | --- | --- | --- | --- | --- | --- |
|  | **France** | **Germany** | **Italy** | **Spain** | **France** | **Germany** | **Italy** | **Spain** | **France** | **Germany** | **Italy** | **Spain** |  |
| **Population (2020)** | 65273511 | 83783942 | 60461826 | 46754778 | 1000000 | 1000000 | 1000000 | 1000000 | (67) | (68) | (69) | (70) |  |
| **DLBCL incidence** | 4842 | 4111 | 4559 | 2301 | 74.2 | 49.1 | 75.4 | 49.2 | Estimation based on HAS DLBCL incidence estimation for  *tisagenlecleucel* and *axicabtagene ciloleucel* assessment (17,18) | Averaged estimation based on IQWiG reviewed DLBCL incidence estimation (range 3143 – 5030) for *tisagenlecleucel* assessment (41) | Estimation based on AIRTUM working group DLBCL incidence estimation for 2015 in Italy after correction for 2015 to 2020 population change (42,69) | Estimation based on GENESIS-SEFH group DLBCL incidence estimation for *axicabtagene ciloleucel* assessment (48) |  |
| **R/R DLBCL population after two therapy lines**  (CAR T-cell therapy EMA label population) | 801 | 661 | 638 | 490 | 12.3 | 7.9 | 10.6 | 10.5 | Estimation based on HAS estimation of relapsed / refractory DLBCL patients after two lines of therapy for *tisagenlecleucel* and *axicabtagene ciloleucel*  (target patient group corresponding to CAR T-cell therapy EMA label population) (17,18) | Averaged estimation based on G-BA estimation of relapsed / refractory DLBCL patients after two lines of therapy for tisagenlecleucel and axicabtagene ciloleucel (target patient group corresponding to CAR T-cell therapy EMA label population; range 502 – 821) (11,12,41)  Note: the G-BA estimations were extrapolated from the statutory health insured population (87.7%) to represent the entire German population (11,12,41) | Estimation based on Belleudi et al. 2021 and Di Rocco et al. 2021 estimation of relapsed / refractory DLBCL patients after two lines of therapy (target patient group corresponding to CAR T-cell therapy EMA label population; 14% of DLBCL patients are refractory or in relapse after second line therapy) (42–44) | Estimation based on GENESIS-SEFH group estimation of relapsed / refractory DLBCL patients after two lines of therapy for *axicabtagene ciloleucel*  (target patient group corresponding to CAR T-cell therapy EMA label population) (49) |  |
| **CAR T-cell therapy eligible DLBCL population** (after two therapy lines) | 481 | 397 | 383 | 294 | 7.4 | 4.7 | 6.3 | 6.3 | Estimation based on HAS estimation of CAR T-cell therapy eligible DLBCL population for *tisagenlecleucel* and *axicabtagene ciloleucel* (on average around 60% of selected patients had been treated in registrational trials) (17,18) | Estimation based on HAS estimation of CAR T-cell therapy eligible DLBCL population for *tisagenlecleucel* and *axicabtagene ciloleucel* (on average around 60% of selected patients had been treated in registrational trials) (17,18) | Estimation based on HAS estimation of CAR T-cell therapy eligible DLBCL population for *tisagenlecleucel* and *axicabtagene ciloleucel* (on average around 60% of selected patients had been treated in registrational trials) (17,18) | Estimation based on HAS estimation of CAR T-cell therapy eligible DLBCL population for *tisagenlecleucel* and *axicabtagene ciloleucel* (on average around 60% of selected patients had been treated in registrational trials) (17,18) |  |
| **CAR T-cell infused DLBCL population (2020)** | 340  (28 pts / mts) | 250  (21 pts / mts) | 110  (9 pts / mts) | 161  (13 pts / mts) | 5.2 | 3.0 | 1.8 | 3.4 | Estimation based on GERS and internal data provided by Kite Pharma Inc./ Gilead Sciences Inc. in personal communication (39)  Estimation is in range of the data reported for the French DESCAR-T registry: 421 DLBCL patients (91% of total 463 patients) that have been infused with CAR T-cell therapies between December 2019 and January 2021 (28), corresponding to 30 patients per month | Estimation based on IQVIA and internal data provided by Kite Pharma Inc./ Gilead Sciences Inc. in personal communication (39)  Estimation is higher than the data reported for the German DRST registry: 323 DLBCL patients (91% of total 356 patients) that have been infused with CAR T-cell therapies between November 2018 and April 2021 in 21 participating centers (26), corresponding to 11 patients per month (or to 15 patients per month if extrapolated to the total 29 German DLBCL CAR T-cell centers in December 2020) (39) | Estimation based internal data provided by Kite Pharma Inc./ Gilead Sciences Inc. in personal communication (39)  Estimation is in range of the data reported for the Italian AIFA registry: 137 DLBCL patients that have been infused with CAR T-cell therapies between August 2019 and December 2020 (37), corresponding to 8 patients per month | Data based on data provided by the Spanish Ministry of Health in personal communication based on a data query submitted (40) |  |

**Supplementary Table 3.** 2020 DLBCL CAR T-cell center density and utilization in France, Germany, Italy, and Spain.

|  | **Absolute numbers** | | | | **Comments / References** | | | |
| --- | --- | --- | --- | --- | --- | --- | --- | --- |
|  | **France** | **Germany** | **Italy** | **Spain** | **France** | **Germany** | **Italy** | **Spain** |
| **Population (2020)** | 65273511 | 83783942 | 60461826 | 46754778 | (67) | (68) | (69) | (70) |
| **CAR T-cell infused DLBCL population (2020)** | 340 | 250 | 110 | 161 | (39) | (39) | (39) | (40) |
| **DLBCL CAR T-cell centers (end of year 2020)** | 24 | 29 | 20 | 9 | (28) (data for January 2021) | (39) (data for December 2020) | (37) (data for December 2020) | (38) (data for December 2020) |
| **DLBCL CAR T-cell centers (per one million inhabitants, 2020)** | 0.37 | 0.35 | 0.33 | 0.19 | calculated | calculated | calculated | calculated |
| **CAR T-cell infused DLBCL patients (averaged per DLBCL CAR T-cell centers, 2020)** | 14.2 | 8.6 | 5.5 | 17.9 | calculated | calculated | calculated | calculated |

**Supplementary Table 4.** Interview questions for participating authors

| **No.** | **Interview question** |
| --- | --- |
| **1** | What are your estimations for the number of DLBCL patients in your country that meet the EMA label requirements? |
| **2** | To your knowledge, what data is available in your country on the number of DLBCL patients that have undergone treatment with licensed CAR T-cell therapies?  *(e.g., registry data, publications, on the number of DLBCL patients registered/ approved, leukapheresed, infused with CAR T-cell therapies)* |
| **3** | If you consider the journey of a DLBCL patient from diagnosis to CAR T-cell infusion, in which key steps do you identify challenges resulting in delays or in challenges to patient access?  *(e.g., patient identification, patient referral, approval of CAR T-cell therapy funding, delivery of CAR T-cell therapy at authorized centers)* |
| **4** | In your opinion, what are the main challenges for DLBCL patient access to CAR T-cell therapies in your country? |
| **5** | What do you consider to be the underlying causes of those challenges? |
| **6** | Considering future developments, what do you expect will become the priority challenges for patient access to CAR T-cell therapies (or to cell and gene therapies with a similar challenge profile)? |
| **7** | Do you have examples of existing best practices for ensuring patient access to CAR T-cell therapies that could represent relevant learnings for the health system? |
| **8** | In your opinion, what should be the ambition of the health system in your country / at European level in respect to CAR T-cell therapies? |
| **9** | What could be potential health system level solutions and coordinated actions to overcome the identified challenges for patient access to CAR T-cell therapies in your country? |

**Additional references**

63. FDA (U.S. Food and Drug Administration). *Kymriah* (2022) <https://www.fda.gov/vaccines-blood-biologics/cellular-gene-therapy-products/kymriah-tisagenlecleucel> [Accessed May 31, 2022]

64. FDA (U.S. Food and Drug Administration). *Yescarta* (2022) <https://www.fda.gov/vaccines-blood-biologics/cellular-gene-therapy-products/yescarta-axicabtagene-ciloleucel> [Accessed May 31, 2022]

65. FDA (U.S. Food and Drug Administration). *Breyanzi* (2022) <https://www.fda.gov/vaccines-blood-biologics/cellular-gene-therapy-products/breyanzi-lisocabtagene-maraleucel> [Accessed May 31, 2022]

66. FDA (U.S. Food and Drug Administration). *Tecartus* (2022) <https://www.fda.gov/vaccines-blood-biologics/cellular-gene-therapy-products/tecartus-brexucabtagene-autoleucel> [Accessed May 31, 2022]

67. Worldometers. *France Population* (2021) <https://www.worldometers.info/world-population/france-population/> [Accessed May 31, 2022]

68. Worldometers. *Germany Population* (2021) <https://www.worldometers.info/world-population/germany-population/> [Accessed May 31, 2022]

69. Worldometers. *Italy Population* (2021) https:// www.worldometers.info/world-population/italy-population/ [Accessed May 31, 2022]

70. Worldometers. *Spain Population* (2021) <https://www.worldometers.info/world-population/spain-population/> [Accessed May 31, 2022]
